# PARP Inhibition by Flavonoids Induced Selective Cell Killing to BRCA2-Deficient Cells

**DOI:** 10.3390/ph10040080

**Published:** 2017-10-12

**Authors:** Cathy Su, Alexis H. Haskins, Chisato Omata, Yasushi Aizawa, Takamitsu A. Kato

**Affiliations:** 1 Department of Environmental and Radiological Health Sciences, Colorado State University, Fort Collins, CO 80523, USA; cathy50720@gmail.com (C.S.); haskinsa@rams.colostate.edu (A.H.H.); hilarious.peeps.v10311028@gmail.com (C.O.); 2 Research and Development Group, Toyo Sugar Refining Co., Ltd., Tokyo 103-0046, Japan; yasushi.aizawa@gmail.com

**Keywords:** quercetin, naringenin, hesperetin, PARP, BRCA2

## Abstract

High consumption of dietary flavonoids might contribute to a reduction of cancer risks. Quercetin and its glycosides have PARP inhibitory effects and can induce selective cytotoxicity in BRCA2-deficient cells by synthetic lethality. We hypothesized that common flavonoids in diet naringenin, hesperetin and their glycosides have a similar structure to quercetin, which might have comparable PARP inhibitory effects, and can induce selective cytotoxicity in BRCA2-deficient cells. We utilized Chinese hamster V79 wild type, V-C8 BRCA2-deficient and its gene-complemented cells. In vitro analysis revealed that both naringenin and hesperetin present a PARP inhibitory effect. This inhibitory effect is less specific than for quercetin. Hesperetin was more cytotoxic to V79 cells than quercetin and naringenin based on colony formation assay. Quercetin and naringenin killed V-C8 cells with lower concentrations, and presented selective cytotoxicity to BRCA2-deficient cells. However, the cytotoxicity of hesperetin was similar among all three cell lines. Glycosyl flavonoids, isoquercetin and rutin as well as naringin showed selective cytotoxicity to BRCA2-deficient cells; hesperidin did not. These results suggest that flavonoids with the PARP inhibitory effect can cause synthetic lethality to BRCA2-deficient cells when other pathways are not the primary cause of death.

## 1. Introduction

Flavonoids are naturally occurring chemicals in plants. They consist of 15 carbon atoms of three rings, two of which are benzene rings connected by a chain with three carbons. Flavonoids present in this form are called aglycones, and can be glycosylated. Quercetin is the aglycone form of a number of other flavonoid glycosides, such as isoquercetin (glycosyl form) and rutin (rhamno-glycosyl form), which are found in citrus fruit, buckwheat and onion [1,2,3]. Naringenin, found in a variety of fruits and herbs, is a bitter flavanone predominant in citrus fruit. Naringin is a naringeinin-7-*O*-glycoside [4]. Hesperetin is a flavanone and hesperidin, hesperetin’s 7-*O*-glycodide, is a natural compound found in lemons and oranges [5]. These flavonoids have a variety of biological activities, including anti-inflammatory, anti-oxidant, free radical scavenging, increase apoptosis rate, lipid peroxidation inhibition effect and cell proliferation inhibition [6,7,8,9]. One of these activities is the inhibition of poly (ADP-ribose) polymerase (PARP), which leads to synthetic lethality for BRCA2-deficient cells [10,11].

Synthetic lethality arises when the combination of two or more genes leads to cell death, but a mutation in only one of these genes does not, and remains viable by itself. Thereby, a synthetic lethality approach to cancer is currently being explored as a means of developing therapies that reduce off-target effects of chemotherapies. Inhibition of PARP can induce synthetic lethality in homologous recombination-deficient cells [12]. Therefore, homologous recombination-deficient BRCA2 homozygous mutant cancer cells are an ideal target for PARP inhibitors [13]. Carriers of BRCA1 or BRCA2 heterozygous mutations have one functional copy of the gene but are highly susceptible to breast and ovarian cancer. Different ethnicities have different heterozygous locus mutations due to founder effects [14,15]. These cancers have homozygous mutations due to loss of heterozygosity. Cancers arising in such people have homozygous mutations, and lack homologous recombination repair. It is known that up to 10% of breast cancers may be caused by inherited mutations in breast cancer susceptibility genes, including BRCA1/2 and PALB2. Mutations in either BRCA gene are associated with a 50–80% elevated lifetime risk of developing breast cancer. For female carriers of the PALB2 mutation, the risk of developing breast cancer has been estimated at 14% by 50 years of age, and 35% by 70 years of age [16]. Mutations in BRCA2 also lead to an increased risk of melanoma, prostate and pancreatic cancer [17].

Although the PARP inhibitory effect of quercetin and its glycosyl flavonoids have been identified in in vitro cell culture [10,11], this inhibitory effect was far lower than clinically available drugs [18]. However, dietary intake of flavonoids included in foods and beverages may be effective at low dosage and with continuous uptake. Several investigators have reported an anti-tumor effect of dietary citrus intake. Citrus extract, or more purified chemicals such as hesperetin, inhibited breast cancer growth [19]. Thus, high citrus fruit intake may be associated with reduced breast cancer risk and recurrence. Two flavanones, heseperetin and naringenin, are common flavonoids in citrus fruit, and they are structurally similar to quercetin. Therefore, we hypothesized that hesperetin and naringenin and their glucosides have PARP inhibitory effects and induce synthetic lethality to BRCA2-deficient cells. This study used Chinese hamster V79 cells origin wild type and BRCA2-deficient cells to present selective toxicity to BRCA2-deficient cells. 

## 2. Materials and Methods 

### 2.1. Chemicals

**Figure 1:**
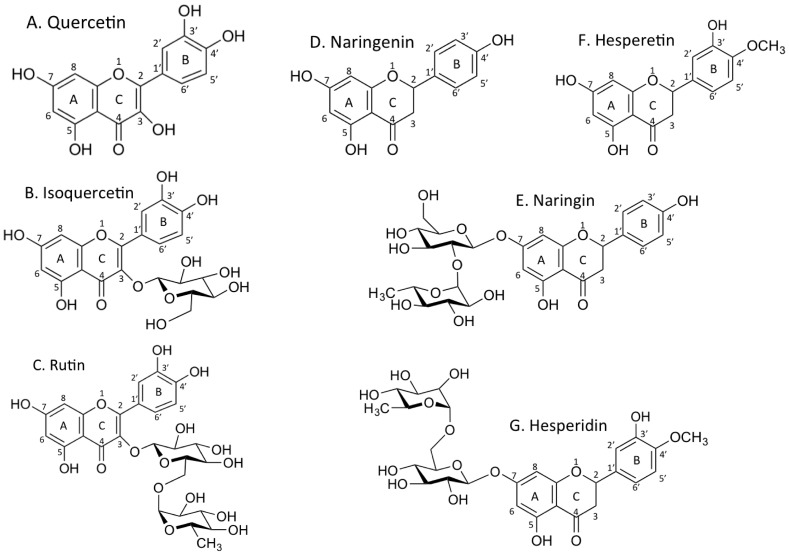
Chemical structures of flavonoids. A. quercetin, B. isoquercetin, C. rutin, D. naringenin, E. naringin, F. hesperetin, G. hesperidin.

Quercetin, isoquercetn, rutin, naringenin, naringin, hesperetin, and hesperidin (Figure 1) were purchased from Sigma-Aldrich (St. Louis, MO, USA). Olaparib, a PARP selective inhibitor (IC_50_ = 5 nM) [20], was purchased from Calbiochem (San Diego, CA, USA). Flavonoids were dissolved in DMSO as 100 mM stock solution and stored at −20 °C.

### 2.2. In Vitro PARP Inhibition Assay

HT Universal Colorimetric PARP Assay Kit (Trevigen, Gaithersburg, MD, USA) was used to assess the capacity of PARP inhibition. Solutions were prepared per the directions provided with the kit from the manufacturer. The histone-coated strip wells were rehydrated using 50 µL of a 1X PARP buffer, and were stored at room temperature. Then, 5 µL of the desired dilutions of the testing compounds and 7.5 µL of PARP enzyme (0.5 unit/well) solution were added to the wells. This was incubated at room temperature for 10 min before the addition of 12.5 µL PARP cocktail, the wells were then left at room temperature for 1 h before washing thoroughly with PBS and PBS with 0.1% Triton X-100. After washing, 50 µL Strep-HRP was added to each well. Again, the wells were incubated at room temperature for 1 h before repeating the washing process. After the wells were washed and dried, by patting on top of a paper towel, 50 µL of TACS-Sapphire was added, and then the wells were placed inside a drawer at room temperature for 15 min before adding 50 µL of 0.2M HCl. The resulting absorbance at 450 nm was read with a Bio-rad benchmark Microplate reader (Bio-Rad, Hercules, CA, USA).

### 2.3. Cell Culture

Chinese hamster V79, BRCA2-deficient mutation V-C8 [21,22], human BRCA2 gene-complemented V-C8 cells were obtained by Dr. Joel Bedford at Colorado State University (Fort Collins, CO, USA). Cells were cultured in heat-inactivated 10% FBS containing alpha-MEM media supplemented with 100 U/mL penicillin, 100 µg/mL streptomycin and 25 ng/mL Amphotericin B at 37 °C. Cells were maintained in the log growth phase.

### 2.4. Cell Survival Asssay

Five hundred cells were plated in P-60 dishes. Two hours after cell plating, a designated amount of flavonoids were added and kept in the incubator for one week. One week later, cells were fixed with 100% Ethanol and stained with 0.1% Crystal Violet solution. In microscopic observation, colonies containing more than 50 cells were designated as survivors. Survival fraction was calculated by dividing the colony number of drug treated samples by the colony number of control samples for each cell line.

### 2.5. Statistics

All experiments were carried out more than three times, independently. Statistical significance was calculated by GraphPad Prism 6 program (GraphPad software, Inc., La Jolla, CA, USA) with two-way ANOVA and multiple comparisons with Tukey test. Multiple comparison-adjusted *p* values lower than 0.05 were regarded as statistically significant.

## 3. Results

### 3.1. Inhibition of PARP Activity

**Figure 2:**
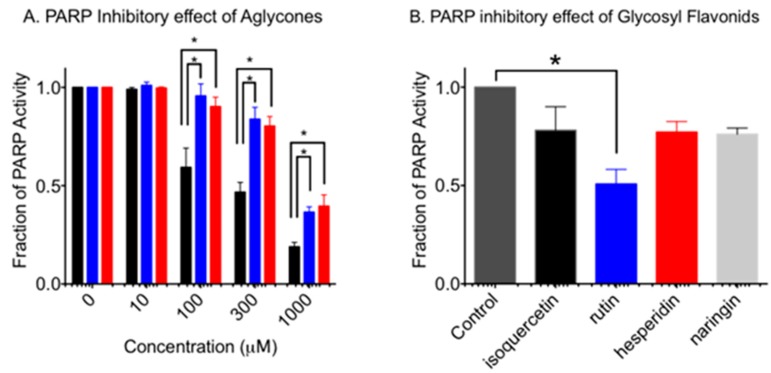
PARP activity inhibition by flavonoids. (**A**) Aglycone form of flavonoids at concentrations between 10 µM and 1000 µM. Black bars indicate quercetin. Blue bars indicate naringenin. Red bars indicate hesperetin. (**B**) Glycosyl form of flavonoids at the concentration of 1 mM. Mean values and standard error of the means from at least three independent experiments are plotted. * indicates statistically significant differences (*p* < 0.05).

PARP activity of aglycone flavonoid was analyzed in vitro (Figure 2A). It was confirmed that quercetin showed significant inhibition at more than 100 µM (*p* < 0.0002). As predicted, it was identified that naringenin and hesperetin showed PARP inhibitory effects and significant inhibition at 1000 µM (*p* < 0.0001). Statistically, quercetin showed the strongest reduction of PARP activity among three aglycone flavonoids at 100 (*p* < 0.0001), 300 (*p* < 0.0001), and 1000 (*p* < 0.001) µM concentrations. Hesperetin and naringenin showed similar PARP inhibition effects (*p* > 0.58).

Previous studies showed a low PARP inhibitory effect for glycosylated quercetin [10]. Therefore, the PARP inhibitory effect of isoquercetin, rutin, naringin, and hesperidin were compared at 1 mM (Figure 2B). Rutin showed a statistically significant PARP inhibitory effect as 0.55 (*p* < 0.0089). Isoquercetin, hesperidin and naringin showed a slight reduction of PARP activity, but this was not statistically significant (*p* > 0.2) compared to the control.

### 3.2. Cytotoxicity to Aglycone Form Flavonoids

**Figure 3:**
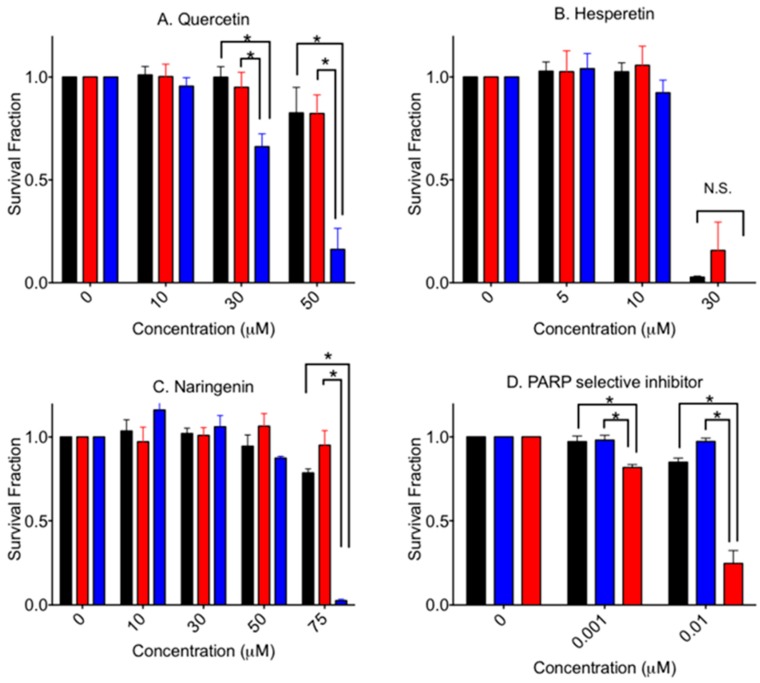
Cytotoxicity by aglycone form of flavonoids and PARP selective inhibitor. (**A**) quercetin, (**B**) hesperetin, (**C**) Naringenin, and (**D**) olaparib. Black bars indicate V79 cells. Red bars indicate V-C8 gene-corrected cells. Blue bars indicate V-C8 BRCA2-deficient cell. Mean values and standard error of the means from at least three independent experiments are plotted. * Indicates statistically significant differences (*p* < 0.05). N.S. means not significant differences.

Figure 3 shows cytotoxicity of an aglycone form of flavonoids in V79, V-C8 and gene-complemented cells. Quercetin showed a statistically significant stronger cytotoxicity to V-C8 cells compared to the V79 and gene-complemented cells. Selective cytotoxicity was clear at 30 µM (*p* < 0.05) and 50 µM (*p* < 0.0001). At 75 µM, gene-complemented cells also showed cellular death (data not shown).

Hesperetin was the most cytotoxic among the tested agents in this study. A 50% cell death value, IC_50_, was approximately 25 µM for V79 cells. There was no significant elevated cytotoxicity for V-C8 cells. On the other hand, naringenin showed a similar cell death pattern compared to quercetin. At 75 µM, V-C8 cells showed statistically significant severe cytotoxicity to naringenin compared to V79 and gene-complemented mutant cells (*p* < 0.001). At 100 µM, no cell lines formed any colonies. Olaparib, a PARP specific inhibitor, showed clear cytoxocicity in BRCA2-deficient cells.

### 3.3. Cytotoxicity to Glycosyl Form Flavonids

**Figure 4:**
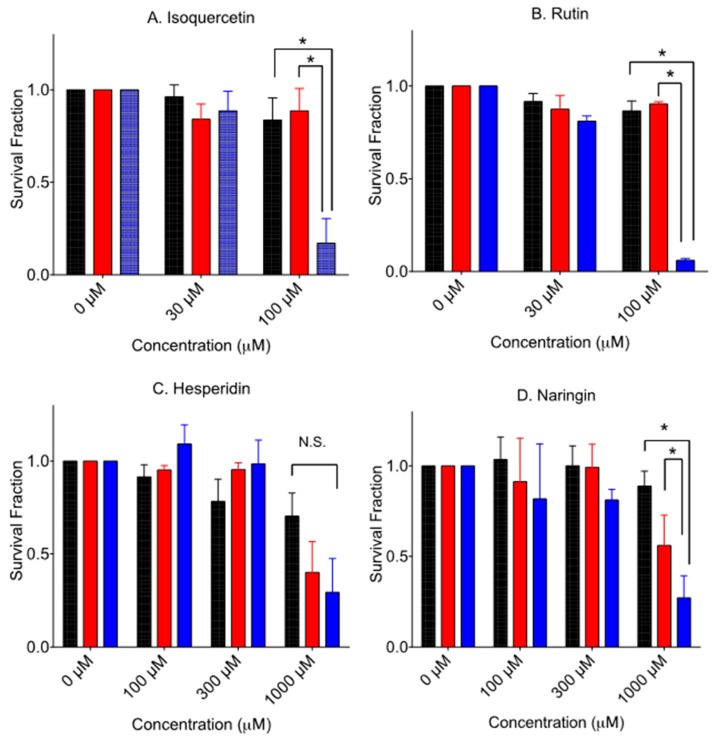
Cytotoxicity by aglycone form of flavonoids. Black bars indicate V79 cells. (**A**) isoquercetin, (**B**) rutin, (**C**) hesperidin, and (**D**) naringin. Red bars indicate V-C8 gene-corrected cells. Blue bars indicate V-C8 BRCA2-deficient cells. Mean values and standard error of the means from at least three independent experiments are plotted. * Indicates statistically significant differences (*p* < 0.05). N.S. means no significant differences.

Figure 4 shows the cytotoxicity of glycosyl forms of flavonoids. Glycosylated flavonoids were less cytotoxic to V79 cells than their aglycone form, as previously reported [10,11]. Isoquercetin and rutin need more than 100 µM to inhibit colony formation for V79 cells. Naringin needs more than 300 µM to inhibit colony formation for V79 cells (data not shown). Contrary to toxic hesperetin, hesperidin needs more than 1000 µM to inhibit colony formation for V79 cells. Selective cytotoxicity of V-C8 cells was observed from 100 µM and 300 µM of isoquercetin, 100 µM of rutin, and 1000 µM of naringin. As seen in hesperetin, hesperidin did not show statistically significant increased death to V-C8 cells compared to V79 cells at 1000 µM exposure.

## 4. Discussion

**Figure 5:**
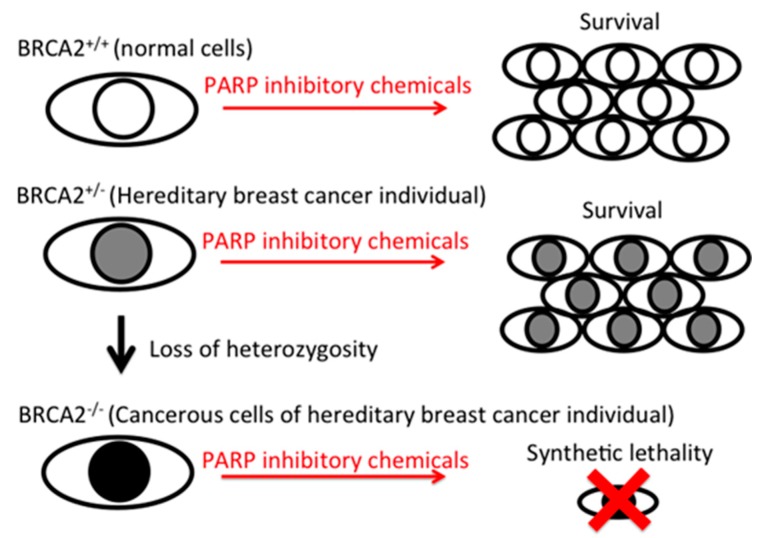
Diagram of PARP inhibitory effect-induced synthetic lethality to BRCA2-deficient cells. BRCA2 normal cells and BRCA2 heterozygous mutated cells can grow in the presence of PARP inhibitory chemicals, but BRCA2 homozygous mutated cells die due to synthetic lethality.

Flavonoids are naturally occurring in plants. Varieties of biological activities were identified and some might explain the potential health benefits from a high intake of flavonoids by one’s daily diet and supplemental intakes. Series of flavonoids have shown anti-cancer effects by anti-proliferation, pro-apoptosis, and anti-inflammatory mechanisms [6,7,8]. The PARP inhibitory effects reported in this study can target specific mutations in any tumor cell, including breast, ovarian, prostate, pancreatic cancers and melanoma [17]. Within breast cancer patients, approximately 10% are heterozygous mutation carriers of the BRCA1/BRCA2/PALB2 gene, and their cancer resulted from the loss of heterozygosity. Since PARP inhibitor-induced synthetic lethality is targeted to homozygous mutant cancer cells and is harmless to heterozygous normal cells, synthetic lethality is a great strategy in controlling tumors without unwanted off-target effects, such as those from chemotherapy (Figure 5).

In addition to the previously reported quercetin, isoquercetin and rutin, this study revealed that naringenin and naringin presented elevated cytotoxicity in BRCA2-deficient cells due to the PARP inhibitory effect (Figure 2, Figure 3 and Figure 4). Since the effective concentrations were much higher than in clinically available drugs (Figure 3), these flavonoids in their natural forms cannot be used for any type of cancer treatment. This implies that further research is needed for the development of a novel PARP inhibitor based on effective flavonoid structures for cancer treatment. However, natural PARP inhibitors that can be readily taken in the form of food or beverage might be beneficial for health, and could be economically advantageous for people when taken as supplements. Quercetin, rich in buckwheat and onion, showed clear PARP inhibitory effects and BRCA2 deficiency specific cytotoxicity (Figure 2 and Figure 4). On the other hand, its glycosyl flavonoids, isoquercetin and rutin also showed similar effects at high concentrations, due to their poor bioavailability [23].

This paper identified that naringenin and naringin, which are rich in orange and grapefruit, also showed similar PARP inhibitory effects and BRCA2 deficiency specific cytotoxicity. However, hesperetin and hesperidin with PARP inhibitory effects did not show BRCA2 deficiency dependent cytotoxicity. Hesperetin was the most toxic chemical among the three aglycone flavonoids tested. Cytotoxicity of hesperetin was approximately three times greater than quercetin and naringenin (Figure 3). In vitro PARP inhibitory effects were observed to be 10 times higher than concentrations causing cytotoxicity (Figure 2). This suggests that PARP inhibition might not be the primary cause leading to cell death. Research showed that hesperetin is known to be a Notch1 activator, which activates the Notch1 signal cascades and suppresses cell proliferation via apoptosis in aplastic thyroid cancer [19,24]. Although two flavanones—naringenin and hesperetin—were not as effective as quercetin in terms of PARP inhibitors and selective cytotoxicity to BRCA2-deficient cells, other flavonoids such as flavone, flavanonol, flavan and flanovol with different phenols may have better PARP inhibitory effects and selective cytotoxicity for BRCA2-deficient cells. 

## 5. Conclusions

In conclusion, this paper identified flavonoids other than quercetin and its glycosides to have PARP inhibitory activity and selective cell toxicity in BRCA2-deficient cells through synthetic lethality. In addition to quercetin, other flavonoids showed glycosylation-reduced PARP inhibitory activity and cellular lethality. Further research should be done to investigate in vivo synthetic lethality-induced chemoprevention and the PARP inhibitory mechanisms of these chemicals.
